# Handgrip weakness, systemic inflammation indicators, and overall survival in lung cancer patients with well performance status: A large multicenter observational study

**DOI:** 10.1002/cam4.5180

**Published:** 2022-09-08

**Authors:** Mengmeng Song, Qi Zhang, Chunhua Song, Tong Liu, Xi Zhang, Guotian Ruan, Meng Tang, Xiaowei Zhang, Hailun Xie, Heyang Zhang, Yizhong Ge, Xiangrui Li, Kangping Zhang, Ming Yang, Qinqin Li, Xiaoyue Liu, Shiqi Lin, Yu Xu, Bo Li, Xiaogang Li, Kunhua Wang, Hongxia Xu, Wei Li, Hanping Shi

**Affiliations:** ^1^ Department of Gastrointestinal Surgery/Clinical Nutrition Capital Medical University Affiliated Beijing Shijitan Hospital Beijing China; ^2^ National Clinical Research Center for Geriatric Diseases, Xuanwu Hospital Capital Medical University Beijing China; ^3^ Key Laboratory of Cancer FSMP for State Market Regulation Beijing China; ^4^ Beijing International Science and Technology Cooperation Base for Cancer Metabolism and Nutrition Beijing China; ^5^ Department of Epidemiology and Statistics Henan Key Laboratory of Tumor Epidemiology College of Public Health, Zhengzhou University Zhengzhou Henan China; ^6^ The Second Affiliated Hospital and Yuying Children's Hospital of Wenzhou Medical University Wenzhou China; ^7^ Liaocheng University Liaocheng China; ^8^ The First Affiliated Hospital of Kunming Medical University Yunnan China; ^9^ Affiliated Hospital of Yunnan University Kunming China; ^10^ Yunnan University Kunming China; ^11^ General Surgery Clinical Medical Center of Yunnan Province Kunming China; ^12^ Department of Nutrition Daping Hospital & Research Institute of Surgery, Third Military Medical University Chongqing China; ^13^ Cancer Center of the First Hospital of Jilin University Changchun China

**Keywords:** ECOG, handgrip weakness, inflammation indicators, lung cancer, prognosis

## Abstract

**Background:**

Systemic inflammation and handgrip weakness have been used to predict mortality in many cancers. The purpose of current study was to evaluate the association of co‐occurrence of inflammation indicators and handgrip weakness with overall survival (OS) of lung cancer (LC) patients with good performance status.

**Methods:**

The cutoff points for handgrip strength (HGS) and the four inflammation indicators were calculated using Maxstat. The time‐dependent receiver operating characteristic curve and C‐index were used to select optimal inflammation indicator for predicting OS of LC patients. The Cox proportional hazard regression model was used to calculate the hazard ratio (HR) of mortality. Kaplan–Meier curves were constructed to evaluate the association of indicators and the OS of LC patients.

**Results:**

Among the 1951 patients, the mean ± standard deviation (SD) age was 60.6 ± 9.9 years, and 1300 (66.6%) patients were male. In patients with good performance status (PS), handgrip weakness (HR, 1.49; 95% confidence interval [95% CI], 1.30–1.70, *p* < 0.001) and low advanced lung cancer inflammation index (ALI) (HR, 2.05; 95%CI, 1.79–2.34, *p* < 0.001), high systemic immune‐inflammation index (SII) (HR, 1.91; 95%CI, 1.66–2.19, *p* < 0.001), high platelet: lymphocyte ratio (PLR) (HR, 1.60; 95%CI, 1.40–1.82, *p* < 0.001), or high neutrophil: lymphocyte ratio (NLR) (HR, 2.01; 95%CI, 1.76–2.30, *p* < 0.001) were associated with increased mortality risk of LC patients. ALI had better C‐index (0.624) and time‐AUC in the prediction of OS in LC patients with good PS than other three combinations. The co‐occurrence of handgrip weakness and low ALI more than doubled the risk of death in LC with good PS (HR, 2.44; 95% CI, 2.06–2.89, *p* < 0.001).

**Conclusion:**

In LC patients who have good PS, patients with combined handgrip weakness and low ALI have the worst prognosis.

**The Trial Registration Number:**

ChiCTR1800020329.

## Graphical abstract

HGS, ALI, SII, NLR, and PLR were independent risk factors for overall survival of lung cancer patients. Co‐occurrence of low HGS and systemic inflammation significantly elevated the risk of death. The predictive ability of handgrip weakness combined with low ALI for OS was better than the other three combinations.

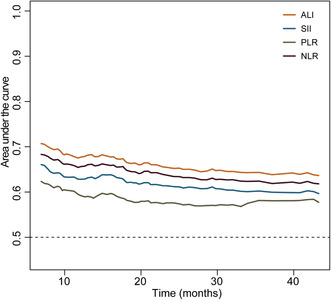

## 1 INTRODUCTION

Lung cancer (LC) is the second most common cancer worldwide and ranks as the leading cause of mortality, accounting for approximately 18% of all cancer deaths.^1^ Although new effective treatment modalities (such as immunotherapy) and advanced diagnostic strategies have developed rapidly, LC mortality remains high.

Due to the close association of functional status of cancer patients with the appropriate therapeutic treatment types,^2^ clinicians generally use certain criteria to assess the health status of patients before adopting treatment measures. Performance status (PS) is an important indicator reflecting patients' general health and functional status and influencing clinical decision‐making.^3^ The Eastern Cooperative Oncology Group (ECOG) scale is widely applied to quantify the performance status of patients with cancer,^4^ and is key to determine the prognosis in several malignant conditions. Tumor‐directed therapy needs to be carefully recommended in solid tumors with a poor PS.^5^, ^6^ Therefore, it is important to find an effective prognostic indicator for patients with different PS to start to triage and choose the most appropriate treatments as well as rehabilitation interventions.

Handgrip strength (HGS) is an effective indicator to measure physical function and performance and has been widely used in observational cohort studies.^7^ HGS, an indicator of overall muscle strength,^8^ can predict survival^9^, ^10^ and is associated with important biological and functional factors, and quality of life in advanced cancer patients.^11^ Accumulated evidence has shown that high level circulating inflammatory markers, such as C‐reactive protein (CRP), TNFα, and interleukin‐6 (IL‐6), are significantly associated with low skeletal muscle strength and mass.^12^, ^13^ As the markers of the systemic inflammatory response (SIR), the advanced lung cancer inflammation index (ALI), neutrophil: lymphocyte ratio (NLR), platelet: lymphocyte ratio (PLR), and systemic immune‐inflammation index (SII) have been proven to be potentially useful biomarkers of LC prognosis.^14^, ^15^ Chronic inflammation affects physical performance, muscle strength, and mass. Patients with increased NLR and PLR values had a higher risk of sarcopenia.^16^ However, not all cases of reduced muscle strength and muscle mass in cancer patients are associated with chronic inflammation. The purpose of our study was to assess the association between low HGS combined with or without chronic inflammation and the overall survival (OS) of LC patients with good PS.

Although handgrip weakness and systemic inflammation have been proven to be prognostic factors of LC and other cancers in previous studies,^9^, ^14^, ^17^, ^18^ most studies mainly illustrate their independent effects in cancer patients. As far as we know, no previous study has assessed the associations between the combinations of host SIR (ALI, SII, NLR, and PLR) and HGS with OS in LC patients. In this study, we evaluated which inflammation indicator was the optimal in the prediction of LC patients with good PS. We further associations between inflammation combined with low HGS and the OS of LC patients with good PS.

## 2 MATERIAL AND METHODS

### 2.1 Participants

Participants were enrolled from the Investigation on Nutrition Status and its Clinical Outcome of Common Cancers (INSCOC) project, which recruited participants from multiple clinical centers across China. In‐person or telephonic questionnaires were used to follow up all participants to obtain relative data on the clinical outcomes of the participants. The inclusion criteria included: (1) ≥18 years old, (2) diagnosis of LC, and (3) length of hospital stay was longer than 48 h. The exclusion criteria included (1) organ transplantation, (2) current pregnancy, (3) HIV infection or AIDS, (4) hospitalized more than twice during the investigation period, and (5) admission to the ICU at the beginning of recruitment. A total of 1951 LC patients were included in current study. All patients enrolled signed informed consent prior to the study, within 48 h of hospital admission. The study protocol was approved by the Institutional Review Committee of Beijing Shijitan Hospital and conformed to the ethical guidelines of the 1975 Declaration of Helsinki.

### 2.2 Collection of baseline data

The baseline information of the patients included age, sex, complications, smoking, the family history of tumor, complications, alcohol consumption, and nutrition support. Each patient underwent a comprehensive interview with a dietitian or clinician conducted of to obtain information of nutrition: the nutritional risk screening 2002 (NRS‐2002) score, Karnofsky Performance Score (KPS), and patient‐generated subjective global assessment (PG‐SGA) score; and anthropometric data: height, HGS, and body weight. Quality of life was evaluated using the European Organization for Research and Treatment of Cancer Quality of Life Questionnaire (EORTC QLQ C30 Version 3.0). The tumor stage for solid tumors was evaluated according to the 8th edition of the American Joint Committee on Cancer (AJCC) TNM staging system.^19^

### 2.3 The measurement of HGS

HGS (non‐dominant hand) is measured using an electronic hand grip dynamometer (CAMARY, Model1 EH101). When measured, the patient seated upright with his arms resting on the armrests and his elbows bent 90 degrees. All patients hold the handle with maximal force for 3 s. Three consecutive tests were performed, and the maximal handgrip strength was recorded.

### 2.4 Assessment of laboratory measurements

Routine blood examinations, including albumin (g/dl), lymphocyte count, neutrophil count, and platelet count, were obtained after at least 9 h of fasting within 24 h of hospitalization. The four inflammation indicators adopted in this study included the ALI (BMI [kg/m^2^] × albumin[g/dl]/NLR), SII (platelet count [×10^9^] × neutrophil count [×10^9^]/lymphocyte count [×10^9^]), PLR (platelet count [×10^9^]/lymphocyte count [×10^9^]), and NLR (neutrophil count [×10^9^]/lymphocyte count [×10^9^]).

### 2.5 Assessment of ECOG PS

The ECOG scale was used to evaluate each patient's PS at enrollment. The ECOG PS were transformed from KPS data according to the criteria as follows^20^: KPS 30–0 (ECOG PS 4), KPS 50–40 (ECOG PS 3), KPS 70–60 (ECOG PS 2), KPS 90–80 (ECOG PS 1), KPS 100 (ECOG PS 0). ECOG 0–1 was classified as the good PS (ECOG 0 or 1), and ECOG 2–4 was defined as poor PS (ECOG≥2). LC patients with good PS comprised the primary research population in current study.

### 2.6 Statistical analysis

All statistical analyses were performed using R (R, Version 4.0.2), involving R packages “survival”, “survminer”, “ggplot2”, “rms”, “timeROC” and “forestplot”, and software SPSS version 21 (SPSS, IBM). Depending on the variable type (continuous or categorical variables; normal distribution or non‐normal distribution), variables were expressed as median (interquartile range, IQR), mean ± standard deviation (SD), or in absolute number and proportion. The optimal cutoff points of HGS for each sex and NLR, ALI, SII and PLR were calculated using the maximally selected rank statistics. The C‐index and time‐dependent receiver operating characteristic (ROC) curves were used to select optimal inflammatory indicator among ALI, NLR, PLR, and SII for predicting the OS of lung cancer. Spearman correlation analysis was performed to evaluate the correlation between HGS and four inflammatory indicators. In addition, we performed a logistic regression on categories of ALI (low or high), PLR (low or high), SII (low or high), and NLR (low or high) as predictors of low HGS (yes or no) to evaluate the association between four inflammatory indicators and low HGS. Cox proportional hazard models were used to assess the relationship of low HGS‐low ALI with OS during follow‐up in patients with good and impaired (ECOG PS 0–1, and ECOG PS 2–4, respectively) PS. HRs and 95% CIs were calculated. Each combination was analyzed independently of other inflammatory markers. Analyses were adjusted for age, sex, TNM, smoking status, alcohol consumption, co‐morbidity, and family history of malignancy. The OS curves were generated using Kaplan–Meier plots, and analyzed using the two‐sided log‐rank test. Finally, stratified analyses were performed to evaluate the main associations of each variable (age, sex, TNM stage, lung cancer types, smoking, alcohol drinking) with survival of LC patients. Two‐sided *p* < 0.05 was considered significant.

## 3 RESULTS

### 3.1 Baseline characteristics

**FIGURE 1:**
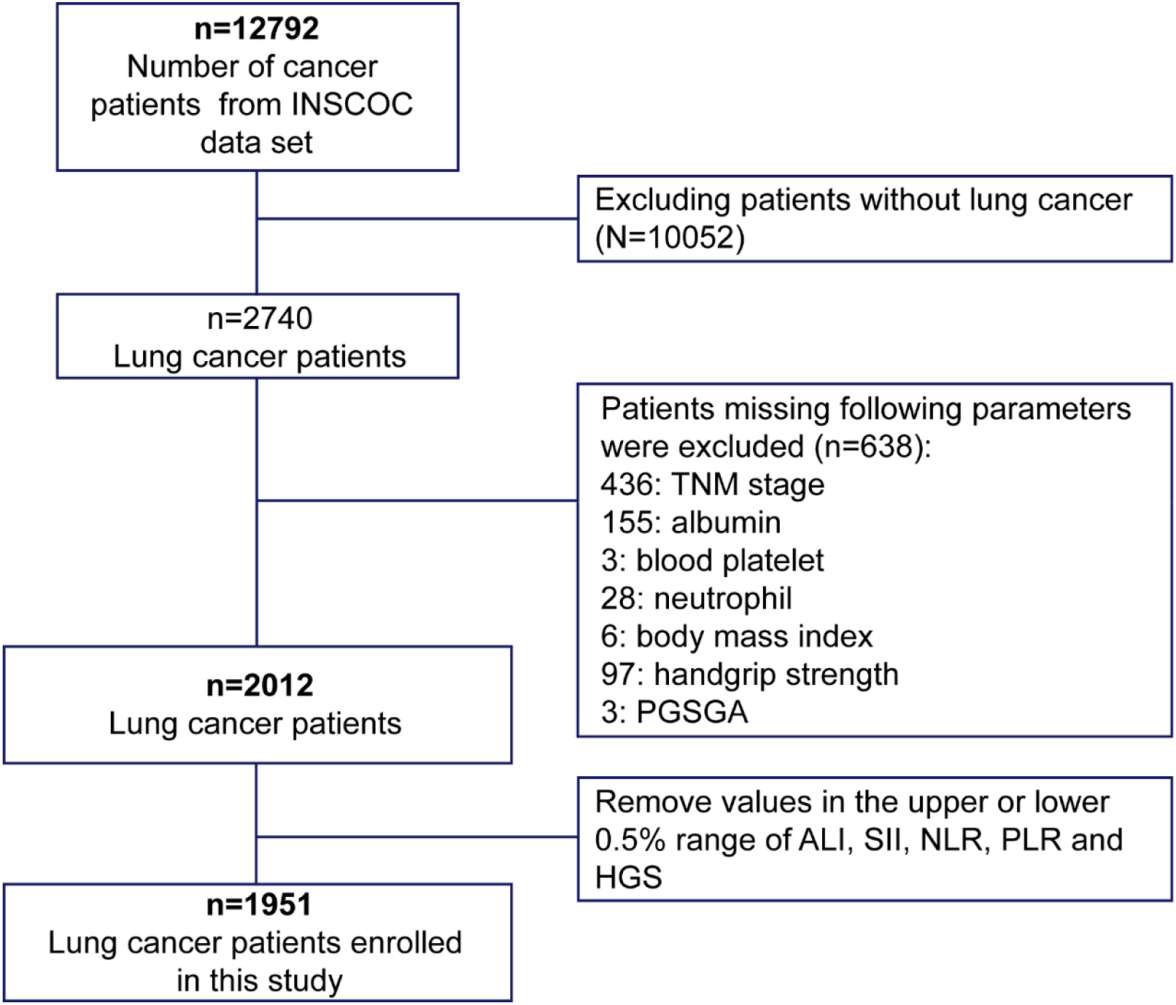
Flow chart. INSCOC, investigation on nutrition status and its clinical outcome of common cancers.

**FIGURE 2:**
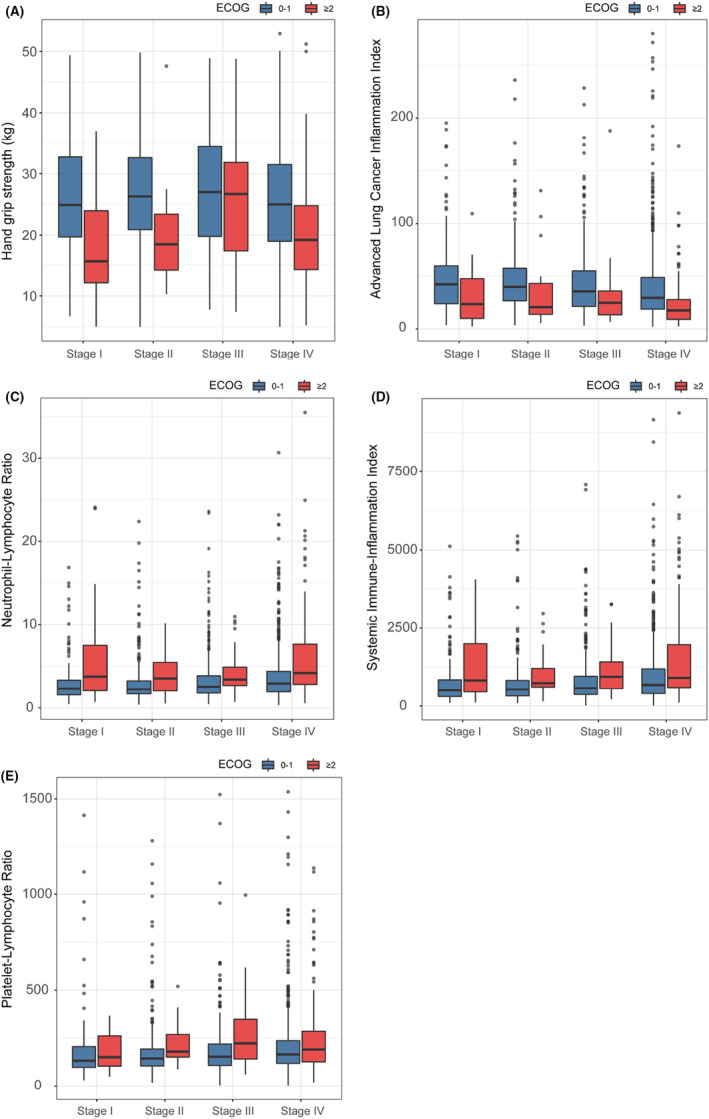
The different levels of (A) HGS, (B) ALI, (C) SII, (D) PLR, and (E) NLR in different ECOG degree stratified by TNM stage. ALI, advanced lung cancer inflammation index; ECOG, Eastern Cooperative Oncology Group; HGS, hand grip strength; NLR, neutrophil lymphocyte ratio; PLR, platelet lymphocyte ratio; SII, systemic immune‐inflammation index.

The selection process of the study participant is shown in Figure 1. After excluding the population with other tumors or with missing data, a total of 1951 patients were selected for subsequent analyses. The baseline characteristics of the patients are summarized in Table 1. The mean age was 60.6 ± 9.9 years, and 1300 (66.6%) were males. Non‐small cell lung cancer (NSCLC) accounted for 80.6%, and small cell lung cancer (SCLC) accounted for 14.7%. The patients with good PS had higher HGS (except for TNM III), higher ALI, lower SII, PLR and NLR (Figure 2) regardless of the TNM stages. Patients with good PS were younger, and were more likely to have higher BMI, and a lower prevalence of co‐morbidity (all *p* < 0.001) compared with patients with a poor PS (Table 1).

**TABLE 1:** Baseline characteristics

	Total (*n* = 1951)	ECOG (0–1) (*n* = 1715)	ECOG≥2 (*n* = 236)	*p*
Age (mean [SD])	60.6 (9.9)	60.1 (9.7)	64.2 (10.7)	<0.001
<65	1252 (64.2)	1121 (65.4)	131 (55.5)	0.004
≥65	699 (35.8)	594 (34.6)	105 (44.5)	
Gender, *n* (%), Male	1300 (66.6)	1138 (66.4)	162 (68.6)	0.532
BMI (mean [SD])	23.0 (3.3)	23.1 (3.2)	21.8 (3.6)	<0.001
<18.5	141 (7.2)	97 (5.7)	44 (18.6)	<0.001
18.5–24	1108 (56.8)	975 (56.9)	133 (56.4)	
≥24	702 (36.0)	643 (37.5)	59 (25.0)	
Histology, *n* (%)				
NSCLC	1572 (80.6)	1388 (80.9)	184 (78.0)	0.399
SCLC	286 (14.7)	249 (14.5)	37 (15.7)	
No histological diagnosis	93 (4.8)	78 (4.5)	15 (6.4)	
TNM				
I	203 (10.4)	179 (10.4)	24 (10.2)	<0.001
II	303 (15.5)	281 (16.4)	22 (9.3)	
III	401 (20.6)	378 (22.0)	23 (9.7)	
IV	1044 (53.5)	877 (51.1)	167 (70.8)	
Complication, *n* (%), Yes	723 (37.1)	613 (35.7)	110 (46.6)	0.002
Smoking, *n* (%) Yes	1167 (59.8)	1027 (59.9)	140 (59.3)	0.925
Alcohol drinking, *n* (%) Yes	492 (25.2)	433 (25.2)	59 (25.0)	0.998
Nutrition support, *n* (%) Yes	219 (11.2)	150 (8.7)	69 (29.2)	<0.001
QLQ‐C30 (mean [SD])	50.7 (9.3)	49.05 (7.77)	62.26 (11.28)	<0.001
PGSGA *n* (%)				
Normal (<4)	986 (50.5)	952 (55.5)	34 (14.4)	<0.001
Malnutrition (≥4)	965 (49.5)	763 (44.5)	202 (85.6)	
Albumin (mean [SD])	38.5 (5.2)	38.91 (4.96)	35.85 (6.12)	
HGS, median (IQR)	24.7 (18.7–31.7)	25.5 (19.5–32.4)	19.2 (14.1–25.3)	<0.001
Male	28.8 (23–35.1)	29.8 (24.0–35.6)	21.3 (17.1–27.0)	<0.001
Female	18.7 (14.3–22.1)	19.1 (15.0–22.3)	14.8 (10.5–18.6)	
ALI, median (IQR)	31.9 (18.9–50.9)	39.3 (36–42.2)	36.3 (32.5–40.2)	<0.001
Low	1053 (54.0)	987 (57.6)	66 (28.0)	<0.001
High	898 (46.0)	728 (42.4)	170 (72.0)	
SII, median (IQR)	640.4 (377.2–1118.4)	604.3 (360.6–1050.0)	886.2 (557.0–1817.6)	<0.001
Low	1380 (70.7)	1251 (72.9)	129 (54.7)	<0.001
High	571 (29.3)	464 (27.1)	107 (45.3)	
PLR, median (IQR)	156.9 (113.1–227.2)	154.1 (111.4–220.9)	189.2 (126.4–286.7)	<0.001
Low	1212 (62.1)	1097 (64.0)	115 (48.7)	<0.001
High	739 (37.9)	618 (36.0)	121 (51.3)	
NLR, median (IQR)	2.8 (1.9–4.345)	2.6 (1.8–4.1)	4.1 (2.7–7.6)	<0.001
Low	1219 (62.5)	1125 (65.6)	94 (39.8)	<0.001
High	732 (37.5)	590 (34.4)	142 (60.2)	

Abbreviations: ALI, Advanced Lung Cancer Inflammation Index; BMI, body mass index; ECOG, Eastern Cooperative Oncology Group; HGS, hand grip strength; NLR, Neutrophil Lymphocyte Ratio; NSCLC, non‐small cell lung cancer; PG‐SGA, Patient‐Generated Subjective Global Assessment; PLR, Platelet Lymphocyte Ratio; SCLC, small cell lung cancer; SII, Systemic Immune‐Inflammation Index.

The cutoff points of HGS in males and females were 31.2 kg and 18.0 kg (Figure S1), respectively. Overall, 54.8% (*n* = 1070) patients showed handgrip weakness at baseline. The cutoff points of ALI, SII, PLR, and NLR were 29.40, 993.78, 184.91, and 3.41, respectively (Figure S1). LC patients with low ALI were older, and had worse PS, lower BMI and HGS, higher NLR, PLR, SII, and QLQ‐C30 scores (all *p* < 0.001) compared with patients with high ALI. There was no significant difference in the distribution of HGS and ALI in histology types (NSCLC and SCLC) (TableS1).

### 3.2 Four inflammation indicators had no association with handgrip weakness

Previous studied reported that inflammation was one cause of handgrip weakness.^21^ To evaluate the association between inflammation indicators and low HGS. We did spearman correlation and found a very weak correlation between four inflammation indicators and HGS (|r| < 0.2) (Figure S2). Then, we regarded low HGS as outcome and did a logistic regression analysis to evaluate the association between inflammation indicators and low HGS. The result showed no associations between ALI (OR, 1.30; 95%CI, 0.93–1.83; *p* = 0.130), SII (OR, 0.85; 95%CI, 0.62–1.17; *p* = 0.325), NLR (OR, 1.37; 95%CI, 0.95–1.97; *p* = 0.088), PLR (OR, 1.07; 95%CI, 0.84–1.35; *p* = 0.578), and the low HGS even after adjustment, respectively (Table S2).

### 3.3 ALI was the better inflammation indicator for predicting OS of patients with LC

**FIGURE 3:**
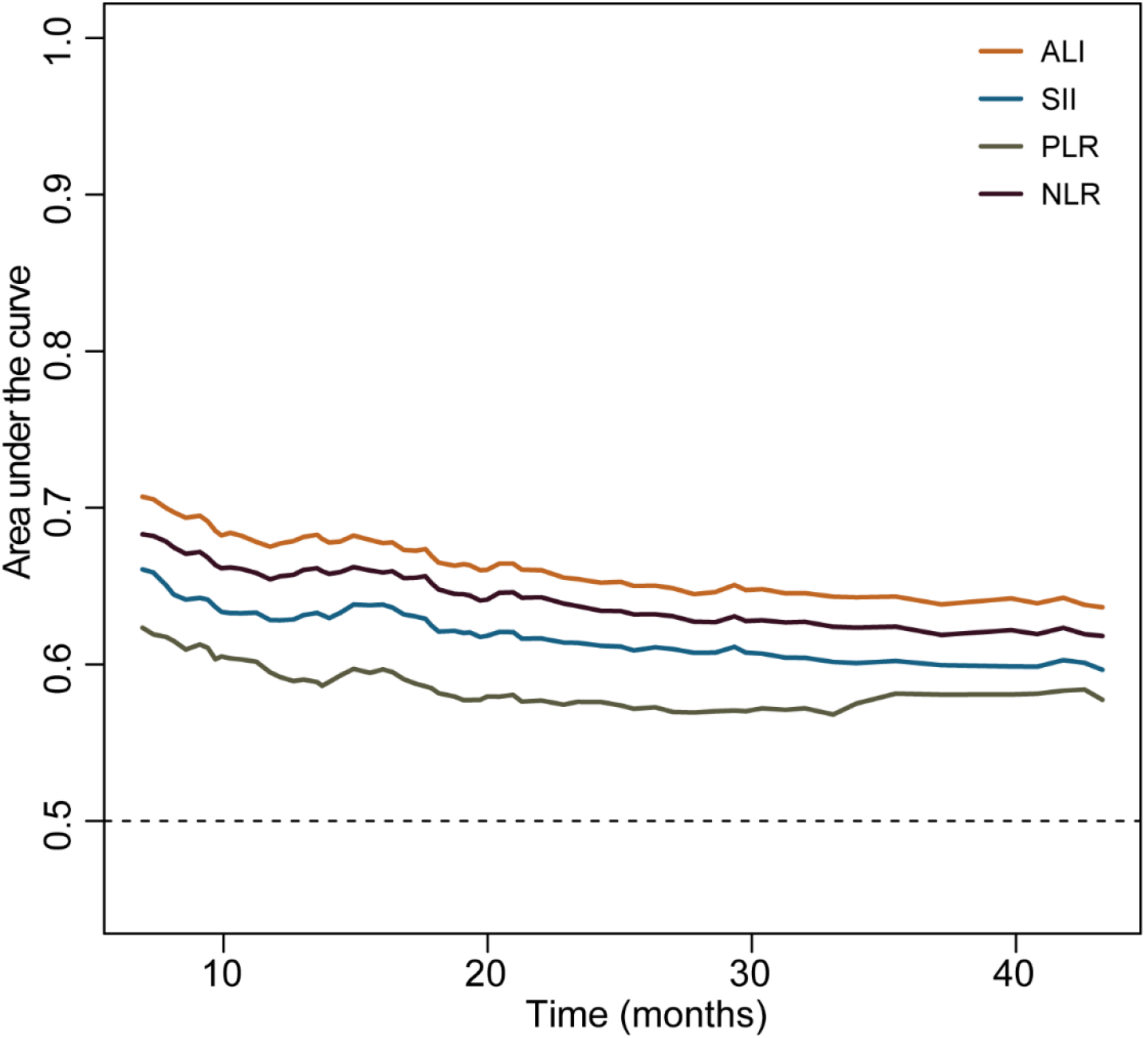
The time‐dependent ROC curve of four predictive inflammation indicators. ALI, advanced lung cancer inflammation index; ECOG, Eastern Cooperative Oncology Group; NLR, neutrophil lymphocyte ratio; PLR, platelet lymphocyte ratio; SII, systemic immune‐inflammation index.

To select the optimal inflammatory indicator for predicting the OS of LC patients with a good PS, we performed a time‐dependent ROC analysis for ALI, SII, PLR, and NLR. The results showed that ALI had the highest AUC among the four inflammatory indicators (Figure 3). In addition, ALI has the highest *C*‐index (0.624; 95%CI, 0.604–0.644) among the four inflammation indicators (Table 2). Therefore, ALI was selected to be optimal inflammation indicator in this study. We further explore the effect of low ALI combined with handgrip weakness on prognosis of different performance status lung cancer patients.

**TABLE 2:** The C‐index of the four inflammatory indicators in lung cancer patients with ECOG 0 or 1

	Overall	
Indicators	C‐index	95%CI
ALI	0.624	0.604–0.644
SII	0.598	0.578–0.619
NLR	0.611	0.591–0.631
PLR	0.570	0.550–0.590

### 3.4 Association between low HGS combined with low ALI and overall survival of patients with LC

#### 3.4.1 All LC patients

During the median follow‐up of 19.1 months, 1063 patients (54.5%) died. The median OS for the whole cohort was 25.6 months. In univariate analysis, low HGS (HR = 1.60, 95%CI:1.41–1.81), low ALI (HR = 2.16, 95%CI:1.92–2.44), high SII (HR = 1.88, 95%CI:1.66–2.13), high PLR (HR = 1.59, 95%CI:1.41–1.80), and high NLR (HR = 2.07, 95%CI:1.83–2.34) were associated with OS of all LC patients (Table 3). In multivariate analysis, low HGS (HR = 1.32, 95%CI:1.17–1.51) or low ALI (HR = 1.85, 95%CI:1.63–2.10) alone was associated with over onefold mortality risk of LC patients. However, low HGS combined with low ALI (HR = 2.44, 95% CI: 2.06–2.89, *p* < 0.001) (Table 3) was associated with over twofold mortality risk of LC patients.

**TABLE 3:** Univariate and Multivariate analysis of Cox proportional hazard models

Groups	Case/control	Total		Case/control	ECOG (0–1)		Case/control	ECOG (≥2)	
		(*N* = 1951)			(*N* = 1715)			(*N* = 236)	
		HR (95%CI)	*p*		HR (95%CI)	*p*		HR (95%CI)	*p*
Univariate analysis									
Low HGS	646/424	1.60 (1.41, 1.81)	<0.001	497/383	1.49 (1.30, 1.70)	<0.001	149/41	1.42 (0.96, 2.09)	0.077
Low ALI	602/296	2.16 (1.92, 2.44)	<0.001	463/265	2.05 (1.79, 2.34)	<0.001	139/31	1.85 (1.30, 2.62)	<0.001
High SII	388/183	1.88 (1.66, 2.13)	<0.001	303/161	1.91 (1.66, 2.19)	<0.001	85/22	1.22 (0.91, 1.64)	0.183
High PLR	473/266	1.59 (1.41, 1.80)	<0.001	378/240	1.60 (1.40, 1.82)	<0.001	95/26	1.16 (0.87, 1.56)	0.309
High NLR	501/231	2.07 (1.83, 2.34)	<0.001	385/205	2.01 (1.76, 2.30)	<0.001	116/26	1.57 (1.15, 2.13)	0.004
Multivariate analysis									
HGS + ALI									
Low HGS		1.32 (1.17, 1.51)	<0.001		1.27 (1.10, 1.46)	<0.001		1.24 (0.81, 1.89)	0.316
Low ALI		1.85 (1.63, 2.10)	<0.001		1.79 (1.57, 2.05)	<0.001		1.60 (1.11, 2.30)	0.012
Multivariate analysis combining low ALI and low HGS									
High HGS + high ALI	224/335	Reference		214/327	Reference		10/8	Reference	
High HGS + low ALI	193/129	1.81 (1.49, 2.19)	<0.001	172/ 122	1.80 (1.47, 2.20)	<0.001	21/7	1.68 (0.78, 3.63)	0.186
Low HGS+ high ALI	237/257	1.30 (1.08, 1.57)	0.006	206/240	1.27 (1.05, 1.55)	0.015	31/17	1.30 (0.62, 2.71)	0.486
Low HGS + low ALI	409/167	2.44 (2.06, 2.89)	<0.001	291/143	2.27 (1.89, 2.74)	<0.001	118/24	2.04 (1.04, 4.00)	0.037

*Note*: Adjusted factors include age, gender, TNM, smoking, alcohol, complication, and family history of tumor.

Abbreviations: ALI, advanced lung cancer inflammation index; ECOG, eastern cooperative oncology group; HGS, hand grip strength; NLR, neutrophil lymphocyte ratio; PLR, platelet lymphocyte ratio; SII, systemic immune‐inflammation index.

#### 3.4.2 LC patients with ECOG PS 0 or 1

The majority of the patients (*n* = 1715, 87.9%) had an ECOG PS of 0 or 1. The median OS was 29.6 months (95%CI: 26.2–32.9 months). A total of 880 (51.3%) patients had low HGS. In univariate analysis, low HGS (HR = 1.49, 95%CI:1.30–1.70), low ALI (HR = 2.05, 95%CI:1.79–2.34), high SII (HR = 1.91, 95%CI:1.66–2.19), high PLR (HR = 1.60, 95%CI:1.40–1.82), and high NLR (HR = 2.01, 95%CI:1.76–2.30) were associated with poor OS of all LC patients (Table 3). In multivariate analysis, both low HGS (HR = 1.27, 95%CI:1.10–1.46) and low ALI (HR = 1.79, 95%CI:1.57–2.05) were independent risk factors of mortality. Importantly, low HGS combined with low ALI had higher risk of mortality compared with those patients with normal HGS and high ALI (HR = 2.27, 95% CI: 1.89–2.74, *p* < 0.001) (Table 3).

#### 3.4.3 LC patients with ECOG PS ≥2

An ECOG PS ≥2 was observed in 239 patients (12.1%). The median OS was 8.8 months. In univariate analysis, only low ALI (HR = 1.85, 95%CI:1.30–2.62) was associated with poor OS of all LC patients (Table 3). In multivariate analysis, low ALI (HR = 1.60, 95%CI:1.11–2.30), but not low HGS (HR = 1.24, 95%CI:0.82–1.89) was independent mortality risk factor of LC. Importantly, only low HGS combined with low ALI had higher risk of mortality compared with those patients with normal HGS and high ALI (HR = 2.04, 95% CI: 1.04–4.00, *p* = 0.037) (Table 3).

### 3.5 LC patients with low HGS combined with low ALI had the worst overall survival (ECOG 0 or 1)

**FIGURE 4:**
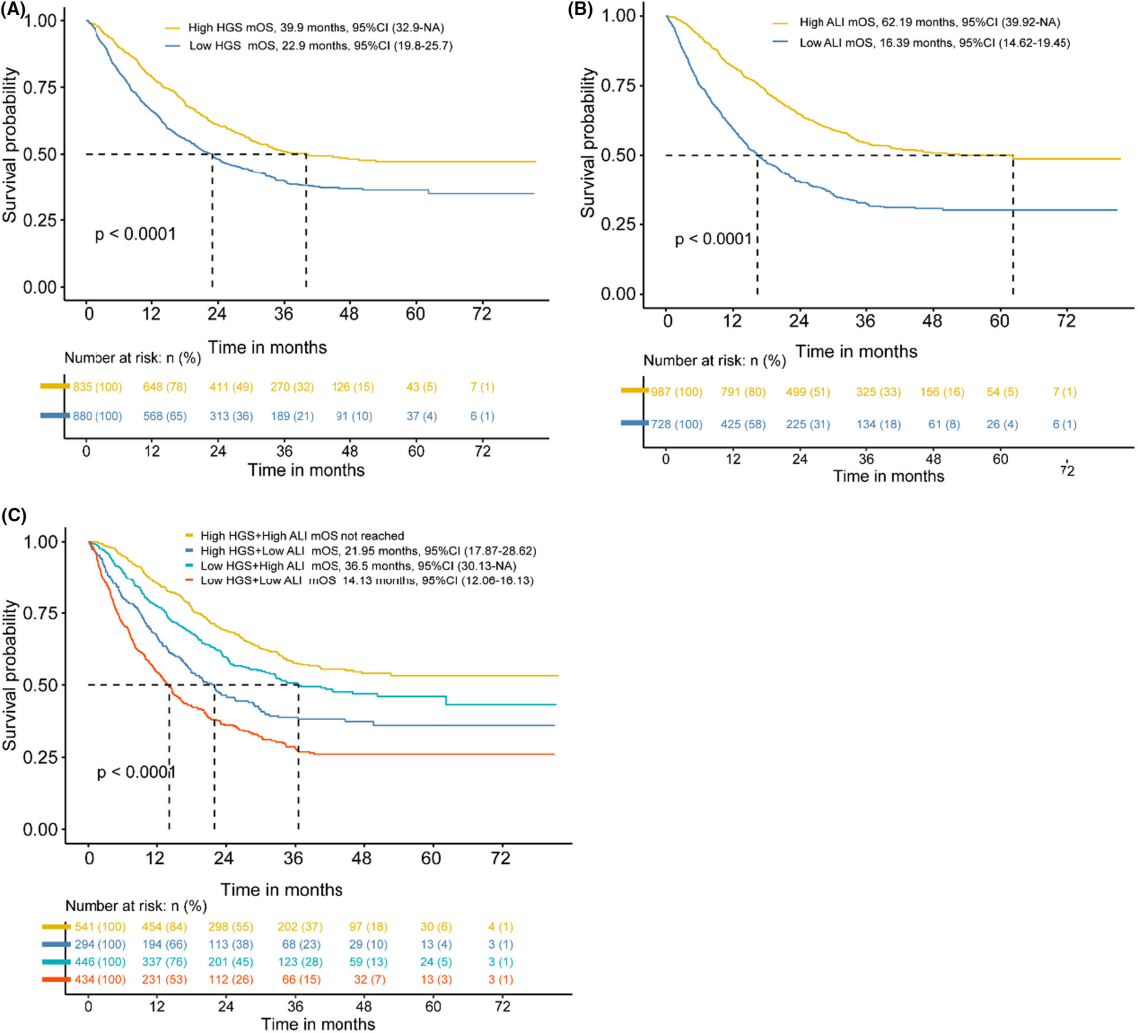
The Kaplan–Meier curves of HGS (A) and ALI (B) and combination of HGS and ALI (C) in lung cancer patients with ECOG 0 and 1. 95%CI, 95% confidence interval; ALI, advanced lung cancer inflammation index; ECOG, Eastern Cooperative Oncology Group; HGS, hand grip strength; mOS, median overall survival; NLR, neutrophil lymphocyte ratio; PLR, platelet lymphocyte ratio; SII, systemic immune‐inflammation index.

As shown in Figure 4A, the median OS (mOS) was significantly shorter in patients with low HGS compared with patients without low HGS. Similarly, the median OS was significantly lower in patients with low ALI (mOS = 16.39 months) than in those with high ALI (mOS = 62.19 months) (Figure 4B). Importantly, the median OS was the shortest in patients with co‐occurrence of low HGS and low ALI (mOS = 14.13 months) compared with high HGS combined with high ALI group (mOS were not reached), high HGS combined with low ALI group (mOS = 21.95 months), and low HGS combined with high ALI group patients (mOS = 36.5 months), respectively (Figure 4C). The Kaplan–Meier curves of low HGS combined with low ALI in different subgroups shown the same results (Figures S3).

### 3.6 Stratified analysis

**FIGURE 5:**
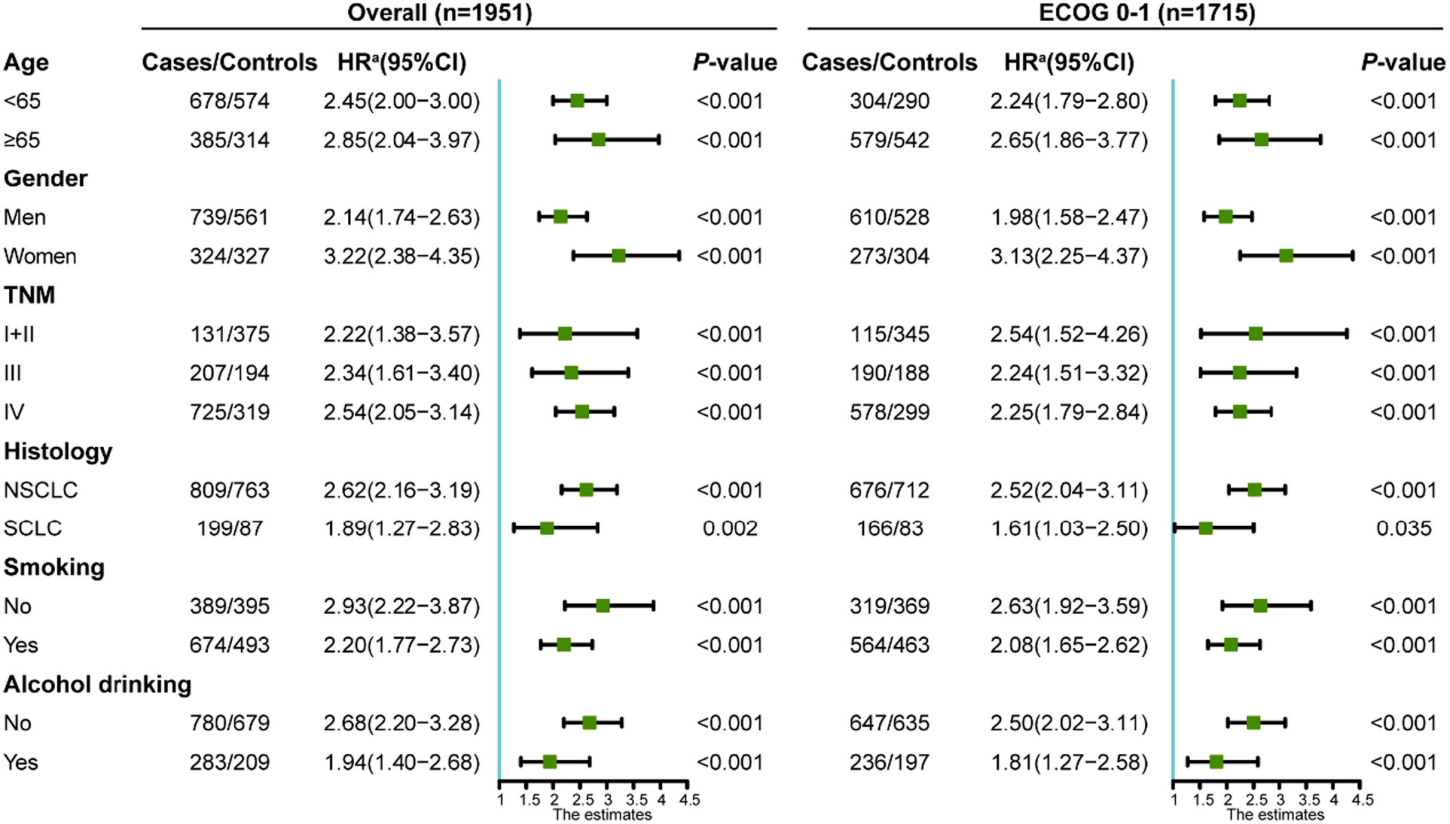
Subgroup analysis of low HGS combined with low ALI in participants with ECOG 0 or 1. The reference group was the patients with high HGS combined with high ALI. Adjusted factors include age, gender, TNM, smoking, alcohol, co‐morbidity, and family history of tumor (excluding the stratified factors). NSCLC, non‐small cell lung cancer; SCLC, small cell lung cancer.

To assess the association between HGS combined with the four inflammation indicators and mortality risk in various subgroup (age, gender, TNM, histology, smoking, and alcohol drinking) patients with good PS, stratified analyses were further performed. For those patients with co‐occurrence of low HGS and low ALI, increased risks of mortality were observed in all subgroups (Figure 5).

## 4 DISCUSSION

To our knowledge, this was a novel study to assess the association between co‐occurrence of inflammatory and low HGS and OS in LC patients. Among 1951 LC patients of our study, 1715 patients had a good PS. Both low HGS and low ALI were independent strong prognostic factors for OS of LC patients. Additionally, our data demonstrated that the co‐occurrence of low HGS and low ALI had doubled mortality risk of LC patients with good PS. If prospectively validated in another large population, handgrip weakness with low ALI may be a suitable combination for identifying patients with high mortality risk, and may aid in timely clinical interventions and improving the prognosis of LC. In addition, handgrip weakness combined with high SII or high NLR could also predict poor OS of LC patients with good PS. However, ALI was a better predictor of the OS of LC patients than other three inflammation indicators. Measures of inflammatory indicators and HGS are easily obtainable in the clinical setting. The co‐occurrence of low HGS and low ALI may represent a kind of “inflammatory low HGS,” a powerful prognostic indicator in LC patients, even those with a good PS.

Previous studies mainly focused on the independent effects of grip strength and inflammatory markers on LC prognosis. Our study suggested that a similar process occurs in LC patients with a good PS regardless of TNM stages at diagnosis, suggesting that handgrip weakness was not only a representation of the advanced stage of the disease. Our research found that inflammatory indicators were not associated with handgrip weakness. However, the co‐occurrence of inflammation and handgrip weakness was associated with an increased mortality risk of LC patients, which was consistent with the previous study on colorectal cancer. For example, pre‐diagnosis inflammation was associated with sarcopenia at the time of diagnosis, and co‐occurrence of sarcopenia and high NLR nearly doubled the risk of death.^22^ In SCLC, a previous study reported that sarcopenic male patients with high NLR had a poor prognosis and intolerance to standard treatment,^23^ which was consistent with our results. Beyond that, HGS was more easily assessed than sarcopenia.

According to statistics, the incidence of male LC was 14.3%, which was higher than that among women (8.4%).^1^ Also in this study, the percentage of male LC patients (66.6%) were also higher than that of the female patients (43.4%). It is well‐known that tobacco smoking is a major cause of LC. In this study, half of the patients were smokers, and more than 88% were male. We found that the overall HGS of smokers was higher than that of non‐smokers. There was no difference between the two groups when the HGS was assessed separately in men and women. The difference in the overall HGS was mainly due to the fact that most of the LC patients who smoked were male (the HGS of male patients was higher than that of females), leading to the illusion that HGS of smokers with LC was higher than that of non‐smokers. In addition, lung cancer patients who smoked also tended to be older, alcohol drinkers, or had lower albumin and ALI. Previous studies have reported that tobacco smoking was immunosuppressive^24^ and strongly associated with low serum albumin levels.^25^ However, low ALI was still an independent risk factor for OS in LC patients after adjusting for tobacco smoking status. Compared with the high ALI group, the LC patients with low ALI tended to be older, malnourished, had a worse quality of life, with a higher percentage of malnutrition, ECOG PS ≥2 or low HGS, and had higher SII, NLR, and PLR.

Intriguingly, we found that LC patients with low HGS had lower ALI, or higher NLR and SII. Higher HGS was found to be associated with lower levels of inflammation, and the association between the HGS and mortality was partly explained by inflammatory indicators.^12^, ^13^, ^21^ Inflammatory processes were also associated with sarcopenia^16^ and muscle atrophy, and thus with weaker grip strength. The inflammatory process in cancer resulted in muscle protein breakdown via reduction of muscle protein synthesis and the activation of ubiquitin proteasomal degradation.^26^, ^27^ However, logistic regression analysis in our study showed that there was no relationship between inflammation and low HGS. In addition, not all muscle mass and strength that decrease were induced by inflammation. Whether the OS of LC patients with low HGS combined with inflammation was worse than that of the patients with low HGS alone remains unclear. Our study revealed that low HGS and four inflammation indicators (ALI, SII, NLR, and PLR) were independent risk factors for OS in LC patients. In addition, LC patients with good PS, and low HGS combined with systemic inflammation had the worse OS compared with those with high HGS or without systemic inflammation. We also demonstrated that LC patients (ECOG PS 0 or 1) with low HGS combined with low ALI or only low ALI had poorer OS than those with only low HGS, suggesting that patients with handgrip weakness but without inflammation had a better outcome. The simultaneous presence of low HGS and low ALI has an additive effect on physical performance. Therefore, this study is of great significance for identifying such patients and providing effective symptomatic treatment to improve the prognosis.

According to a pooled analysis of the International Lung Cancer Consortium, underweight patients (BMI <18.5 kg/m^2^) account for approximately 4% of LC cases.^28^ Consistent with previous studies, approximately 7% of patients were underweight (with BMI < 18.5 kg/m^2^) in this study, while overweight and obese patients (BMI ≥24 kg/m^2^) accounted for a larger proportion (36.0%). A previous study has found a nonlinear positive association between grip strength and BMI.^29^ We found that 40.1% of LC patients with good PS had low HGS, suggesting that they were likely to have sarcopenic obesity. The sarcopenic obesity, with a risk of synergistic complications from both sarcopenia and obesity, is a high‐risk geriatric syndrome predominantly.^30^ Thus, ALI combined with grip strength can help better identify patients without reduced BMI who would still need attention and timely intervention. The co‐occurrence of low HGS and low ALI can better predict the poor prognosis of LC patients with well PS.

There were several strengths in this current study. First, it may be the largest study to examine the association between systemic inflammation biomarkers and low HGS in LC. The strength of this study comes from its multi‐center, prospective nature, and large sample size; therefore, there is a good representation of the population. Second, HGS is noninvasive, fast, cost‐efficient, and easy to implement, which are very important in the oncology domain. Third, our study included all TNM stages, despite the fact that the number of patients with TNM stage I was relatively small.

However, limitations in this study should also be noticed. Similar to previous studies,^22^ our study cannot unravel the relationship between inflammation, HGS, and cancer‐associated outcomes. HGS and inflammation were measured at the same time, thus reflecting only their correlation with them. CRP was not included in this study because of the limited available data, thus only four inflammation indicators were included. Further studies shall be performed to assess more available inflammation indicators when an adequate number of participants are enrolled in the study.

## 5 CONCLUSION

In LC patients with good PS, handgrip weakness and low ALI, high NLR, high SII, and high PLR were independent prognostic indicators. We also found that the co‐occurrence of handgrip weakness and inflammation more than doubled risk of mortality in LC patients with good PS. It may represent a kind of “inflammatory low HGS.” Compared with other three inflammation indicators, ALI is a better inflammatory indicator combined with low grip strength to predict prognosis of LC. The combination of the two indicators provides effective prognostic stratification for lung cancer patients with well PS, and could provide guidance for the treatment and nursing of LC patients. However, further study needs to be performed to confirm this result.

### Headings

- HGS, ALI, SII, NLR, and PLR were independent risk factors for overall survival of lung cancer patients.
- Among these four indicators, ALI was optimal inflammation indicator to predict the prognosis of lung cancer patients with well PS.
- Co‐occurrence of low HGS and systemic inflammation significantly associated with the elevated the mortality risk of LC patients with well PS.

## AUTHOR CONTRIBUTION

Hanping Shi conceptualized, supervised, and made funding acquisition; Mengmeng Song contributed to methodology, software, Writing –original draft preparation; Qi Zhang contributed to methodology and software; Chunhua Song validated, investigated, did writing –reviewing and editing; Tong Liu and Xi Zhang did writing –reviewing and Editing; Guotian Ruan validated; Xiangrui Li investigated; Yizhong Ge did methodology and investigation; Xiaowei Zhang, Kangping Zhang, Ming Yang, Qinqin Li: Investigation, Shiqi Lin, Yu Xu, Bo Li, and Xiaogang Li investigated; Kunhua Wang, Hongxia Xu, and Wei Li did investigation and data curation.

## CONFLICT OF INTEREST

All authors have completed the ICMJE uniform disclosure form. The authors have no conflicts of interest to declare.

## ETHICS APPROVAL

This study was performed in line with the principles of the Declaration of Helsinki. Approval was granted by the Institutional Review Committee of Beijing Shijitan Hospital.

## CONSENT TO PARTICIPATE

Informed consent was obtained from all individual participants included in the study.

## Supporting information


Table S1

Figure S1

## Data Availability

The datasets generated during and/or analyzed during the current study are available from the corresponding author on reasonable request.
