# Supplementary material for: Handgrip weakness, systemic inflammation indicators, and overall survival in lung cancer patients with well performance status: A large multicenter observational study
Source: Cancer Med. 2022 Sep 8;12(3):2818–30. doi: 10.1002/cam4.5180 (PMC9939150; doi:10.1002/cam4.5180)
Supplement: Supplementary file 1 — Table S1 [file CAM4-12-2818-s001.docx]

**TableS1 Baseline characteristics stratified by HGS and ALI.**

|  | High HGS (n=881) | Low HGS (n=1070) | *P* | High ALI (n=1053) | Low ALI (n=898) | *P* |
| --- | --- | --- | --- | --- | --- | --- |
| Age, median (IQR) | 59.0 (52.0-64.0) | 63.0 (56.0-69.0) | <0.001 | 60.0 (53.0-66.0) | 62.0 (55.0-69.0) | <0.001 |
| Gender, n (%) |  |  |  |  |  |  |
| Male | 511 (58.0) | 789 (73.7) | <0.001 | 681(64.7) | 619(68.9) | 0.052 |
| Female | 370 (42.0) | 281 (26.3) |  | 372(35.3) | 279(31.1) |  |
| BMI, median (IQR) | 22.9 (20.975-25.1) | 22.7 (20.6-24.9) | <0.001 | 23.7 (21.6-25.7) | 21.8 (19.9-23.9) | <0.001 |
| <18.5 | 31 (3.5) | 110 (10.3) | <0.001 | 41(3.9) | 100(11.1) | <0.001 |
| 18.5-24 | 446 (50.6) | 662 (61.9) |  | 525(49.9) | 583(64.9) |  |
| ≥24 | 404 (45.9) | 298 (27.9) |  | 487(46.2) | 215(23.9) |  |
| TNM |  |  |  |  |  |  |
| I | 108 (12.3) | 95 (8.9) | 0.001 | 134 (12.7) | 69 (7.7) | <0.001 |
| II | 149 (16.9) | 154 (14.4) |  | 208 (19.8) | 95 (10.6) |  |
| III | 195 (22.1) | 206 (19.3) |  | 239 (22.7) | 162 (18.0) |  |
| IV | 429 (48.7) | 615 (57.5) |  | 472 (44.8) | 572 (63.7) |  |
| Histology |  |  |  |  |  |  |
| NSCLC | 720 (81.7) | 852 (79.6) | 0.419 | 856 (81.3) | 716 (79.7) | 0.610 |
| SCLC | 123 (14.0) | 163 (15.2) |  | 161 (15.3) | 125 (13.9) |  |
| No histological diagnosis | 38 (4.3) | 55 (5.1) |  | 36 (3.4) | 57 (6.3) |  |
| Smoking, n (%) |  |  |  |  |  |  |
| No | 409 (46.4) | 375 (35.0) | <0.001 | 441 (41.9) | 343 (38.2) | 0.108 |
| Yes | 472 (53.6) | 695 (65.0) |  | 612 (58.1) | 555 (61.8) |  |
| Complication, n (%) |  |  |  |  |  |  |
| No | 583 (66.2) | 645 (60.3) | 0.008 | 657(62.4) | 571(63.6) | 0.619 |
| Yes | 298 (33.8) | 425 (39.7) |  | 396(37.6) | 327(36.4) |  |
| Alcohol drinking, n (%) |  |  |  |  |  |  |
| No | 681 (77.3) | 778 (72.7) | 0.023 | 782(74.3) | 677(75.4) | 0.604 |
| Yes | 200 (22.7 | 292 (27.3) |  | 271(25.7) | 221(24.6) |  |
| Nutrition support, n (%) |  |  |  |  |  |  |
| No | 802 (91.0) | 930 (86.9) | 0.005 | 966(91.7) | 766(85.3) | <0.001 |
| Yes | 79 (9.0) | 140 (13.1) |  | 87(8.3) | 132(14.7) |  |
| QLQ-C30, median (IQR) | 49.26 (8.22) | 51.79 (10.00) | <0.001 | 47 (43-53) | 50 (45-58) | <0.001 |
| PGSGA n (%) |  |  |  |  |  |  |
| Normal | 550 (62.4) | 436 (40.7) | <0.001 | 626(59.4) | 360(40.1) | <0.001 |
| Malnutrition | 331 (37.6) | 634 (59.3) |  | 427(40.6) | 538(59.9) |  |
| ECOG, n (%) |  |  |  |  |  |  |
| 0-1 | 835 (94.8) | 880 (82.2) | <0.001 | 987(93.7) | 728(81.1) | <0.001 |
| ≥2 | 46 (5.2) | 190 (17.8) |  | 66(6.3) | 170(18.9) |  |
| Albumin, median (IQR) | 40.1 (36.8-42.9) | 38.2 (34.4-41.2) | <0.001 | 40.5 (37.6-43.1) | 36.8 (33-40.3) | <0.001 |
| HGS, median (IQR) | 20.8 (16.2-27.2) | 27.6 (21.3-33.7) | <0.001 | 25.8 (19.7-33.3) | 23.6 (17.8-30.1) | <0.001 |
| Male | 29.6 (21.9-35.7) | 28.8 (23.2-34.7) | 0.578 | 30.4 (24.7-36.9) | 26.7 (21.2-32.2) | <0.001 |
| Female | 18.7 (14.2-22.0) | 18.5 (14.5-22.4) | 0.846 | 19.5 (15.2-22.8) | 17.8 (13.2-2) | <0.001 |
| ALI, median (IQR) | 37.4 (23.3-56.3) | 27.7 (16.3-46.8) | <0.001 | 48.9 (37.9-67.0) | 17.8 (11.9-23.6) | <0.001 |
| SII, median (IQR) | 577.9 (346.0-955.4) | 700.7 (418.5-1196.0) | <0.001 | 417.9 (275.3-590.6) | 1158.9 (768.1-1815.3) | <0.001 |
| PLR, median (IQR) | 149.6 (109.0-218.5) | 162.1 (116.5-235.6) | <0.001 | 127.7 (97.8-170.0) | 209.8 (149.7-286.5) | <0.001 |
| NLR, median (IQR) | 2.5 (1.7-3.9) | 3.1 (2.0-4.7) | <0.001 | 1.91 (1.5-2.4) | 4.5 (3.6-6.5) | <0.001 |

Chi-Squared Test and Wilcoxon rank test were used for the comparation of continues and category variables respectively. The patients without histological diagnosis were not included in the analysis of type subgroup. NSCLC, non-small cell lung cancer; SCLC, small cell lung cancer; BMI, body mass index; PG-SGA, Patient-Generated Subjective Global Assessment; ALI, Advanced Lung Cancer Inflammation Index; SII, Systemic Immune-Inflammation Index; PLR, Platelet-Lymphocyte Ratio; NLR, Neutrophil-Lymphocyte Ratio; HGS, hand grip strength; ECOG, Eastern Cooperative Oncology Group.

**Table S2** Association of additional markers of systemic inflammation with handgrip weakness at lung cancer (n=1951).

|  | Odds ratio for handgrip weakness (95%CI) | *P* |
| --- | --- | --- |
| Advanced Lung Cancer Inflammation Index |  |  |
| ≥29.41 | Reference | 0.130 |
| <29.41 | 1.3 (0.93,1.83) |  |
| Systemic Immune-Inflammation Index |  |  |
| <993.78 | Reference | 0.325 |
| ≥993.78 | 0.85 (0.62,1.17) |  |
| Neutrophil-Lymphocyte Ratio |  |  |
| <3.41 | Reference | 0.088 |
| ≥3.41 | 1.37 (0.95,1.97) |  |
| Platelet-Lymphocyte Ratio |  |  |
| <184.91 | Reference | 0.578 |
| ≥184.91 | 1.07 (0.84,1.35) |  |

Adjusted factors include age, gender, TNM, smoking, alcohol, co-morbidity and tumor family history. 95%CI, 95% confidence interval.
