# Supplementary material for: Handgrip weakness, systemic inflammation indicators, and overall survival in lung cancer patients with well performance status: A large multicenter observational study
Source: Cancer Med. 2022 Sep 8;12(3):2818–30. doi: 10.1002/cam4.5180 (PMC9939150; doi:10.1002/cam4.5180)
Supplement: Supplementary file 2 — Figure S1 [file CAM4-12-2818-s002.docx]

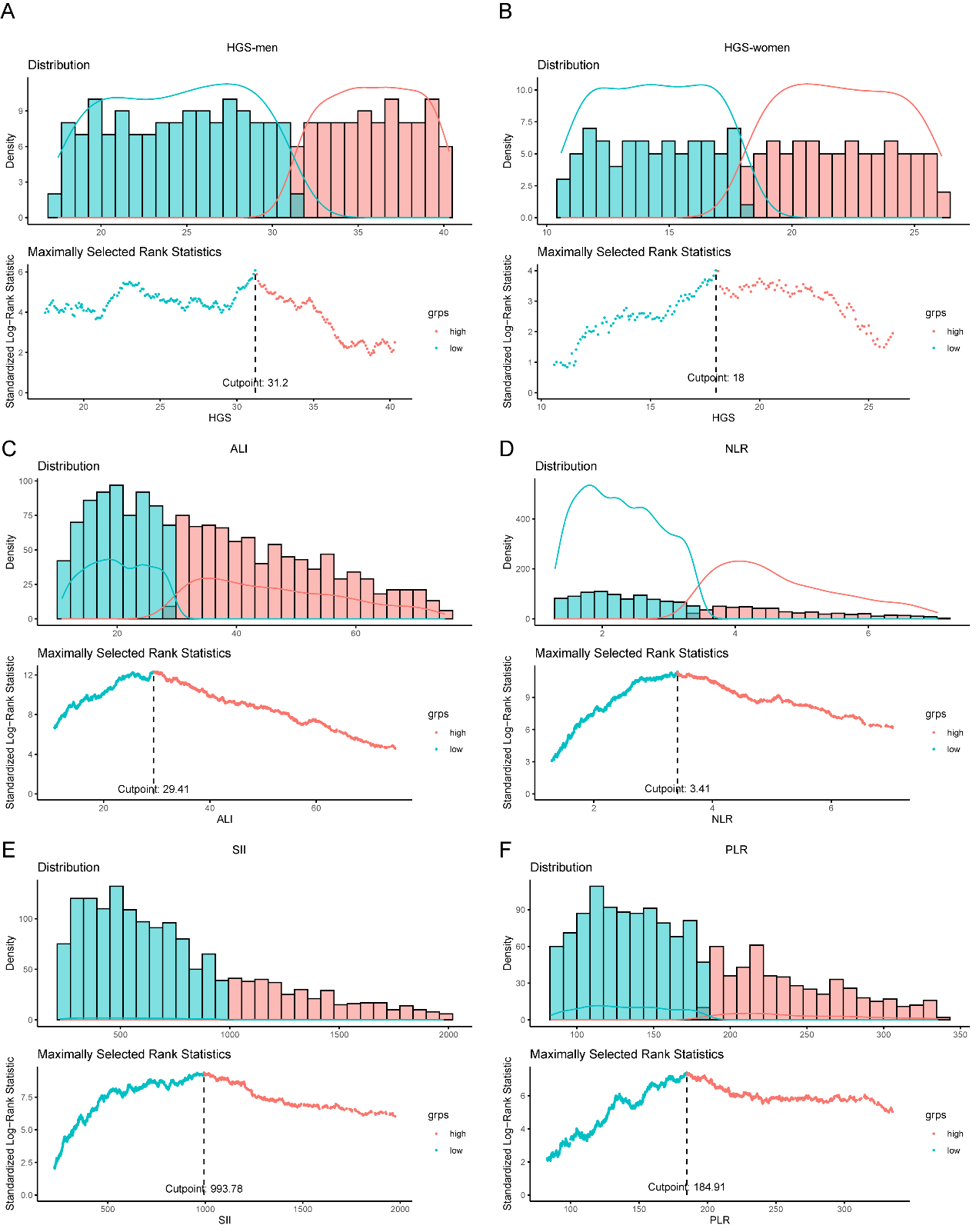


**Figure S1 The cutoff points of HGS (male and female), ALI, SII, NLR and PLR.**

ALI, advanced lung cancer inflammation index; SII, systemic immune-inflammation index; PLR, platelet-lymphocyte ratio; NLR, neutrophil-lymphocyte ratio; HGS, hand grip strength; ECOG, Eastern Cooperative Oncology Group.


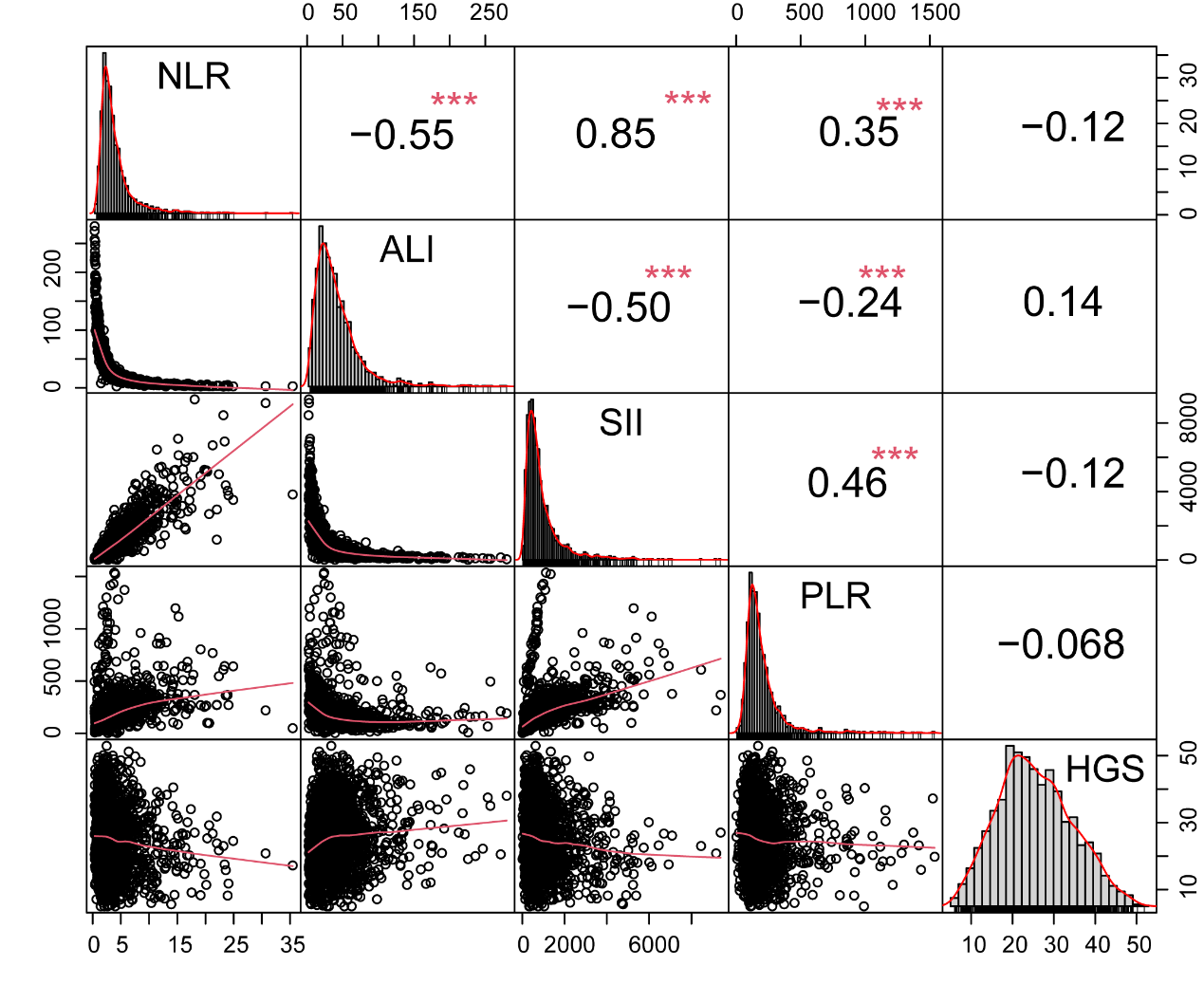


**Figure S2 The visualized correlation analysis of HGS, ALI, SII, PLR and NLR in patients with lung cancer.** Correlation coefficient: 0.8-1.0, very strong correlation; 0.6-0.8, strong correlation; 0.4-0.6, moderate correlation; 0.2-0.4：weak correlation; 0.0-0.2, very weak correlation or no correlation. HGS, hand grip strength; ALI, advanced lung cancer inflammation index; PLR, platelet-lymphocyte ratio; NLR, neutrophil to lymphocyte ratio.

**
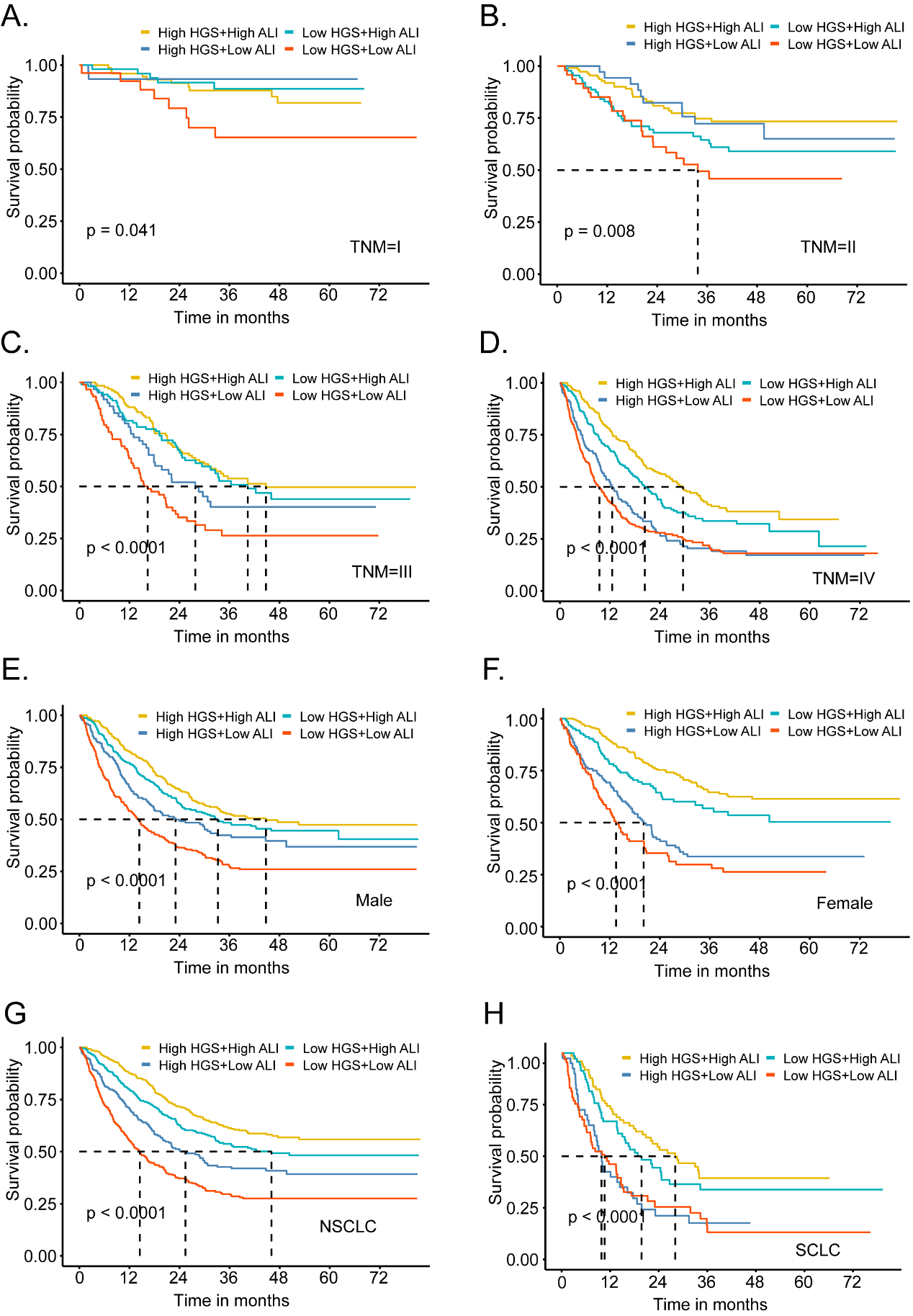
**

**Figure S3. The Kaplan-Meier curves of HGS combined with ALI stratified by TNM stages, sex and pathology types in lung cancer patients with ECOG 0 and 1.**

ALI, Advanced Lung Cancer Inflammation Index; ECOG, Eastern Cooperative Oncology Group.
